# Totally percutaneous endovascular repair for ruptured abdominal aortic aneurysms

**DOI:** 10.3389/fsurg.2022.1040929

**Published:** 2022-10-21

**Authors:** Shirli Tay, Mohamed S. Zaghloul, Mehreen Shafqat, Chao Yang, Kshitij A. Desai, Gayan De Silva, Luis A. Sanchez, Mohamed A. Zayed

**Affiliations:** ^1^ Section of Vascular Surgery, Department of Surgery, Washington University School of Medicine, St. Louis, MO, United States; ^2^ Department of Vascular Surgery, Union Hospital, Tongji Medical College, Huazhong University of Science and Technology, Wuhan, China; ^3^ Division of Molecular Cell Biology, Washington University School of Medicine, St. Louis, MO, United States; ^4^ McKelvey School of Engineering, Department of Biomedical Engineering, Washington Univesrity, St. Louis, MO, United States; ^5^ Department of Surgery, Veterans Affairs St. Louis Health Care System, St. Louis, MO, United States

**Keywords:** ruptured abdominal aortic aneurysm, endovascular aortic aneurysm repair, percutaneous, femoral access, emergent procedure

## Abstract

**Purpose:**

The PEVAR Trial demonstrated that compared to open femoral exposure, elective percutaneous endovascular AAA repair (ePEVAR) is associated with decreased perioperative morbidity and access site complications. We hypothesized that PEVAR for ruptured AAA (rPEVAR) may also improve perioperative morbidity compared to open femoral exposure (rEVAR). There are currently no reports that evaluate the utility and outcomes of rPEVAR.

**Materials and methods:**

From 2015 to 2021, all patients who underwent an endovascular repair of a ruptured AAA at a single institution were included in the study and grouped into rPEVAR and rEVAR. Demographics, procedural details (successful preclose technique, conversion to femoral cutdown), postoperative variables (blood transfusion, ICU and hospital length of stay) and short-term outcomes (30-day major adverse events (30-day MAE) and 30-day femoral access-site complications (30-day FAAC)) were collected and compared with 50 historical ePEVAR patients from the PEVAR Trial. Statistical significance was determined using *χ*^2^ or Fisher's exact test for categorical variables, and Mann–Whitney *U*-test for continuous variables.

**Results:**

35 patients were identified (21 rPEVAR; 14 rEVAR), 86% were male with a mean age of 72 ± 9 years. All patients underwent emergent endovascular aortic repair with 100% technical success. Seventeen patients (49%) presented with evidence of hemorrhagic shock and 22 patients (63%) had blood transfusion. 30-day MAE occurred in 12 patients (34%) (7 rPEVAR; 5 rEVAR). There was no difference in demographic, perioperative outcomes and 30-day MAE rate between rPEVAR and rEVAR patients. Compared to ePEVAR patient (from PEVAR trial), rPEVAR patients had higher rate of 30-day MAE (34% vs. 6%; *p *< 0.006) but no difference in 30-day FAAC (19% vs. 12%; *p *= 0.54). The success rate of the preclose technique was higher in ePEVAR compared to rPEVAR (96% vs. 76%; *p *= 0.02), but the rate of conversion to femoral cutdown was similar between the two groups (10% vs. 4%; *p *= 0.57).

**Conclusion:**

Emergent rPEVAR appears to have similar outcomes when compared to rEVAR. Although patients undergoing rPEVAR have higher 30-day major adverse events rate compared to ePEVAR, the method of percutaneous femoral cannulation does not appear to increase the overall procedural or 30-day femoral artery access-site complications.

## Introduction

Progressive abdominal aortic aneurysm (AAA) expansion can lead to rupture, hemorrhagic shock, and death (1, 2). It is estimated that the incidence of ruptured AAA is approximately 1%–3% in men >65 years old, and in 70%–95% this can lead to a fatal event (1, 3–5). Thus in addition to timely diagnosis of ruptured AAA, timely repair is essential for preventing severe morbidity and death.

Endovascular aortic aneurysm repair (EVAR) has become the standard of care for elective repair of AAAs over the last two decades, and is associated with relatively low perioperative morbidity and mortality (2, 6). The common femoral artery (CFA) is the default arterial access site for introduction of the aortic endograft for EVAR. Totally percutaneous EVAR (PEVAR) was first introduced in 1999 (7), and refers to the practice of CFA cannulation site closure using a “preclose” technique with a Perclose ProGlide® (Abbott Vascular) closure device. Since then, there has been wide adoption of this technique (8), and in 2013 an industry sponsored, multicenter, randomized controlled trial of PEVAR was preformed (9). This demonstrated that elective percutaneous endovascular AAA repair (ePEVAR) was associated with less perioperative morbidity and access site complications compared to traditional open cut-down exposure of the CFA.

Despite the growing experience with ePEVAR, there are currently no reports that evaluate the utility of percutaneous techniques for EVAR for the treatment of ruptured AAA (rPEVAR). To evaluate the feasibility and outcomes of rPEVAR using Perclose ProGlide closure device, we sought to retrospectively review our experience at our medical center between 2015 and 2020. Patient perioperative variables were retrospectively reviewed and compared to 50 historical ePEVAR patients from the PEVAR Trial that used the same ProGlide® CFA closure device.

## Materials and methods

### Study design

This was a retrospective study of consecutive patients between January 2015 to January 2020 who presented to our medical center with ruptured AAA and underwent emergent endovascular repair. No patients were excluded from our analysis during this study period. Patients who underwent EVAR through percutaneous femoral access were grouped into rPEVAR group, and those who underwent EVAR through open femoral artery access were grouped into rEVAR group. Patient demographic characteristics including co-morbidities, surgical intraoperative variables, and postoperative variables were all evaluated. Additionally, 50 historical patients in the PEVAR Trial that received identical femoral arterial closure with the Perclose ProGlide technique were also included in our comparative analysis as part of the ePEVAR group (9).

### Perclose proGlide insertion technique

All rPEVARs were performed with the suture-mediated Perclose ProGlide closure system by 5 surgeons at our medical center (10–12). To perform this, local anesthetic was injected into the subcutaneous tissue in the bilateral groins over the anticipated course of the CFAs. One centimeter stab oblique incisions were created in the bilateral groins and blunt finger dissection is used to separate the underlying subcutaneous tissue, ether pre- or post-ultrasound (US) guided cannulation of the CFA with a micropuncture needle. The access was typically performed at least 1 cm proximal to the origin of the profunda femoris artery and below the level of the inguinal ligament. A 6 or 7Fr sheath dilator was then used to dilate the cannulation track, and routinely two perclose ProGlide devices were deployed in 3 and 10 o'clock orientations. No less than two ProGlide devices were used in each groin access site. Care was taken to not to pull on the remaining ProGlide sutures and were secured with clamps for closure at the end of the operation.

### rPEVAR procedure

**Figure 1:**
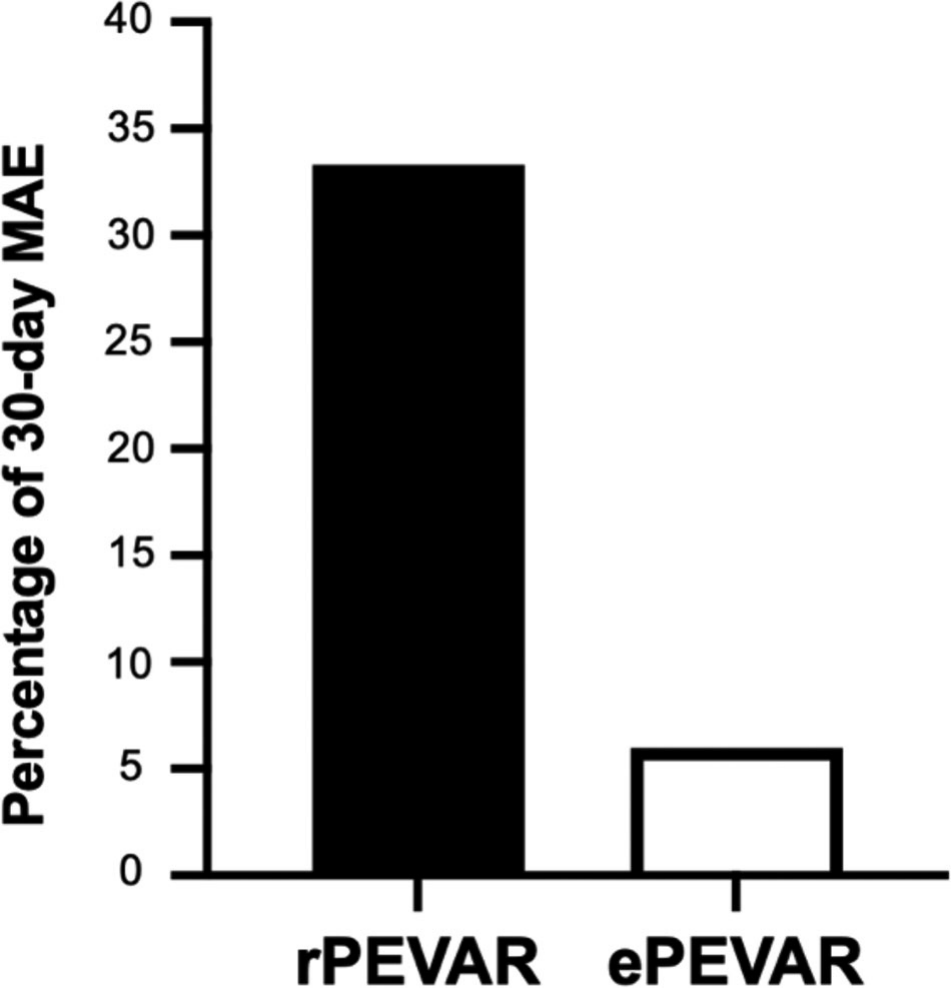
Relative percentage difference in 30-day major adverse events (MAE) between patients that underwent rPEVAR versus ePEVAR.

After successful percutaneous cannulation and deployment of ProGlide devices in the bilateral CFAs, soft wires were then advanced from the CFAs to the infrarenal aorta using an MPA catheter. Wires were then exchanged for a stiff Lunderquist wire that was advanced to the proximal descending thoracic aorta. A 12Fr 45 cm sheath was advanced to the perivisceral aorta from the contralateral CFA cannulation site. An aortic occlusion Coda balloon was advanced through the sheath and inflated in the distal thoracic aorta. At this point the patient was given a bolus of intravenous heparin (50–100 U/kg). If a staged anesthetic plan was used, general anesthesia was then administered and the patient was endotracheally intubated. If the patient remained hemodynamically unstable the Coda balloon would then be maintained inflated until EVAR completion. An aortogram was then performed to confirm anatomic feasibility of EVAR and intraoperative measurements were obtained for proper aortic endograft selection. EVAR was then performed in the standard fashion with temporary switching of the Coda balloon placement from the ipsilateral CFA to the contralateral CFA during advancement of the endograft mainbody. Following deployment of the endograft iliac limbs, completion angiogram was routinely performed for all procedures to confirm ruptured AAA seal and patency of visceral arteries, renal arteries, and iliac artery systems (Figure 1).

### Femoral arteriotomy closure

After EVAR, a soft wire are advanced into the iliac artery and the femoral sheaths were retracted into the distal iliac arteries. Retrograde sheathograms were performed to confirm adequate patency and integrity of the bilateral iliac artery systems. The sheaths were then sequentially removed and the femoral cannulation sites were closed one at a time using the pre-deployed perclose ProGlide devices at the initial point of cannulation as previously described (10–12). Post-closure pharmacological heparin reversal with protamine sulfate was administered and manual pressure was maintained over the cannulation site for at least 10 min and until hemostasis was confirmed.

### Postoperative management

All patients were transferred to an intensive care unit (ICU) for postoperative care. Postoperative computed tomography angiography (CTA) was performed following EVAR at 1 month. Follow-up angiographic imaging was typically performed at 6 and 12 months following the operation, then annually thereafter.

### Clinical outcomes

Technical success, associated complications, and major adverse events were evaluated at approximately 1 month. We defined closure technical success as complete hemostasis after the procedure without vascular complications such as formation of arteriovenous fistula, femoral neuropathy, hematoma, arterial dissection, infection, lymphocele, thrombosis/occlusion with loss of Doppler signal, vascular injury requiring groin re-exploration, acute lower extremity ischemia attributed to arterial access requiring intervention, or access-related bleeding requiring transfusion. Procedural success was defined as successful delivery and placement of endograft without major adverse events or vascular complications. Major adverse events included death, conversion to open repair, bowel ischemia, cardiac-related morbidity, neurological complications, renal failure, and secondary procedure for type I/III endoleak. Additional in-hospital outcomes such as procedure time, ICU length of stay, hospital length of stay, and any blood transfusion requirements were also evaluated.

### Statistical analysis

Study variables were analyzed using STATA software version 17.0. All continuous variables were reported as mean ± standard deviation. To compare study groups, categorical and continuous variables were evaluated using *χ*^2^ or Fisher's Exact test, and Mann–Whitney *U*-test, respectively. All tests were two-sided and *p *< 0.05 was considered statistically significant.

## Results

### Patient demographics

Between January 2015 and January 2020, 35 patients with ruptured AAA underwent emergent endovascular repair. Ten patients (29%) had a preoperative CTA and all patients had an intraoperative aortogram. All patients were found to have adequate infrarenal aortic neck diameters and lengths to accommodate an EVAR. Thirty patients (86%) were male with a mean age of 72 ± 9 years and BMI of 30 ± 6 kg/m^2^ (Table 1). The majority of patients had a history of hypertension (86%) and hyperlipidemia (54%), while 16 patients (46%) had a history of coronary artery disease (Table 1). Two patients (6%) previously had a myocardial infarction and 4 patients (11%) received prior percutaneous coronary interventions (Table 1). As expected with ruptured AAA, the majority of patients (49%) were admitted with hemorrhagic shock (Table 1).

**Table 1:** Baseline characteristics of the rPEVAR and rEVAR patients.

	rPEVAR (*n* = 21)	rEVAR (*n* = 14)	Total (*n* = 35)	*p*
Age (years)	70 ± 8	75 ± 10	72 ± 9	*0* *.* *12*
Male sex	18 (86)	12 (86)	30 (86)	*1* *.* *00*
Height (cm)	177 ± 8	179 ± 8	177 ± 8	*0* *.* *49*
Weight (kg)	93 ± 21	96 ± 25	95 ± 22	*0* *.* *85*
BMI (kg/m^2^)	30 ± 7	30 ± 6	30 ± 6	*0* *.* *98*
Smoking	8 (38)	5 (36)	13 (37)	*0* *.* *89*
Diabetes	6 (29)	4 (29)	10 (29)	*1* *.* *00*
MI	1 (5)	1 (7)	2 (6)	*1* *.* *00*
CAD	9 (43)	7 (50)	16 (46)	*0* *.* *68*
Prior PCI	4 (19)	0	4 (11)	*0* *.* *13*
CHF	4 (19)	3 (21)	7 (20)	*1* *.* *00*
HLD	12 (57)	7 (50)	19 (54)	*0* *.* *68*
HTN	18 (86)	12 (86)	30 (86)	*1* *.* *00*
COPD	7 (33)	5 (36)	12 (34)	*0* *.* *88*
Hemorrhagic shock	10 (48)	7 (50)	17 (49)	*1* *.* *00*
TAA	1 (5)	0	1 (3)	*1* *.* *00*
CVA	0	0	0	–
Renal failure	0	0	0	–

rPEVAR, ruptured percutaneous EVAR; rEVAR, ruptured EVAR with femoral cutdown; BMI, body mass index; CVA, cerebrovascular accident; COPD, chronic obstructive pulmonary disease; CHF, congestive heart failure; CAD, coronary artery disease; PCI, percutaneous coronary intervention; MI, myocardial infarction; TAA, thoracic aortic aneurysm. Data are presented as number (%) for categorical variables and mean ± standard deviation for continuous variables.

Twenty-one patients (60%) underwent rPEVAR, and 14 patients (40%) underwent rEVAR (without the Perclose ProGlide closure system) (Table 1). We observed no differences in the presenting demographics between the two patient groups (Table 1). When compared to the historical ePEVAR patients, we observed a lower incidence of smoking (37% vs. 86%; *p *< 0.001) and hyperlipidemia (54% vs. 90%; *p *< 0.001) among rPEVAR group, but the remaining baseline characteristics were otherwise similar between the two groups (Supplementary Table S1).

### Perioperative and short-term outcomes

We achieved a 100% technical procedural and treatment success rates in both the rPEVAR and rEVAR (study) groups compared to 94% and 88%, respectively, in the ePEVAR control group. We did not observe any significant difference in the perioperative outcomes between rPEVAR and rEVAR patients and the incidence rate of 30-MAE was similar between them (33% vs. 36%; *p *= 0.88) (Table 2).

**Table 2:** Perioperative and short-term outcomes of the rPEVAR and rEVAR patients.

	rPEVAR (*n* = 21)	rEVAR (*n* = 14)	Total (*n* = 35)	*p*
**Procedural and in-hospital outcomes**				
Procedure time (mins)	136 ± 61	260 ± 13	157 ± 73	*0*.*06*
Blood transfusion	11 (52)	11 (79)	22 (63)	*0*.*16*
ICU length of stay (h)	96 ± 128	137 ± 180	113 ± 150	*0*.*30*
Hospital stay (days)	16 ± 28	11 ± 11	14 ± 23	*0*.*60*
**Major adverse events at 30 days**				
30-day MAE	7 (33)	5 (36)	12 (34)	*0*.*88*
Death	2 (10)	0	2 (6)	*0*.*51*
Bowel ischemia	1 (5)	1 (7)	2 (6)	*1*.*00*
Cardiac morbidity	2 (10)	1 (7)	3 (9)	*1*.*00*
Neurologic complication	1 (5)	2 (14)	3 (9)	*0*.*55*
Renal failure	3 (14)	1 (7)	4 (11)	*0*.*64*
Respiratory complication	3 (14)	2 (14)	5 (14)	*1*.*00*
Secondary procedure	6 (29)	2 (14)	8 (23)	*0*.*43*

rPEVAR, ruptured percutaneous EVAR; rEVAR, ruptured EVAR with femoral cutdown; ICU, Intensive Care Unit; MAE, major adverse events. Data are presented as number (%) for categorical variables and mean ± standard deviation for continuous variables.

**Figure 2:**
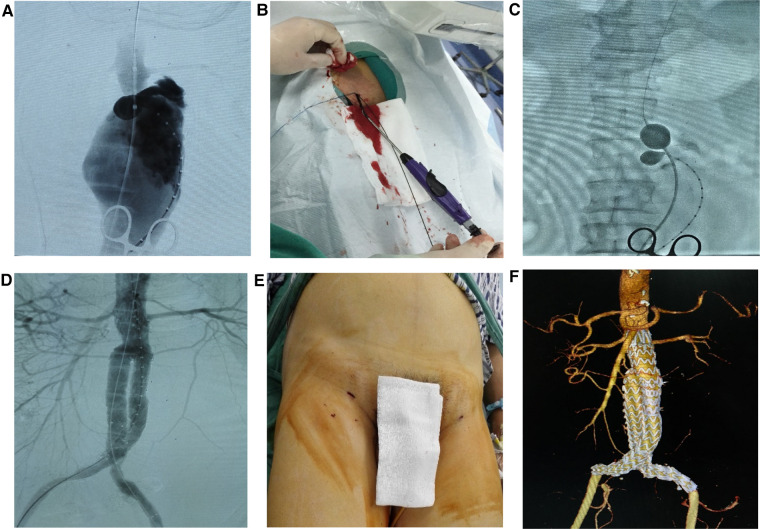
Ruptured percutaneous (rPEVAR) operative procedure (**A**) angiogram of ruptured AAA. (**B**) Percutaneous cannulation and deployment of ProGlide devices in the bilateral CFAs. (**C**) A stiff Lunderquist wire and a12Fr 45 cm sheath were advanced to the perivisceral aorta then a Coda balloon was advanced through the sheath and inflated in the distal thoracic aorta. (**D**) EVAR was performed. (**E**) The sheaths were removed and the femoral cannulation sites were closed using the pre-deployed ProGlide devices. (**F**) Follow-up 3D CTA 30-day post EVAR.

In the study group, we also observed significantly longer average procedure times (by 50 min), ICU length of stay (by 3.6 days), and hospital length of stay (by 12.7 days), compared to ePEVAR (Supplementary Table S2). Blood transfusion was needed in 22 (63%) of study patients, compared to 4 (8%) of ePEVAR patients (Supplementary Table S2). In addition, patients in rPEVAR group had a significantly higher rate of 30-day MAE compared to ePEVAR (34% vs. 6%; *p *< 0.006; Figure 2). However, compared rEVAR, rPEVAR showed no significant difference in the procedure times (260 ± 13 vs. 136 ± 61 min; *p* = 0.06, respectively; Table 2).

### Femoral access-site techniques and complications

Patients who underwent rPEVAR had no significant difference in 30-FAAC when compared to ePEVAR patients (19% vs. 12%; *p *= 0.54) (Table 3). The rate of access-site infection was significantly higher in rPEVAR to ePEVAR (14% vs. 0%; *p *= 0.023); other access-site complications were otherwise similar between the two groups (Table 3). Regarding access-site technique, the success rate of the preclose technique was higher in ePEVAR compared to rPEVAR (96% vs. 76%; *p* ≤ 0.02; Table 3). However, the rate of conversion to femoral cutdown was low, and not significantly different between ePEVAR and rPEVAR (10% vs. 4%; *p* ≤ 0.57; Table 3).

**Table 3:** Femoral access-site techniques and complications.

	rPEVAR (*n* = 21)	ePEVAR* (*n* = 50)	Total (*n* = 71)	*p*
**Procedural access technique**				
Successful preclose	16 (76)	48 (96)	64 (90)	***0***.***02***
Conversion to femoral cutdown	2 (10)	2 (4)	4 (5)	*0*.*57*
**Femoral access-site complications at 30 days**				
30-day FAAC	4 (19)	6 (12)	10 (14)	*0*.*054*
Hematoma	1 (5)	0	1 (1)	*0*.*29*
Dissection	1 (5)	0	1 (1)	*0*.*29*
Infection	3 (14)	0	3 (4)	***0***.***023***
Thrombosis/occlusion	0	2 (4)	2 (3)	*1*.*00*
Vascular injury	0	1 (2)	1 (1)	*1*.*00*
Lower extremity ischemia	0	2 (4)	2 (3)	*1*.*00*
Bleeding/transfusion	1 (5)	1 (2)	2 (3)	*0*.*50*
Arteriovenous fistula	0	0	0	*–*
Femoral neuropathy	0	0	0	*–*
Lymphocele	0	0	0	*–*

^*^ ePEVAR, elective percutaneous EVAR from PEVAR trial; rPEVAR, ruptured percutaneous EVAR; rEVAR, ruptured EVAR with femoral cutdown; FAAC, Femoral Artery Access Complications. Data are presented as number (%) for categorical variables and mean ± standard deviation for continuous variables.

## Discussion

Prior experience demonstrates that PEVAR is a safe and feasible option for femoral cannulation during elective EVAR (11, 13–15). However, since operative urgency and efficiency are considerably more pressing in the setting of ruptured AAAs, it is of interest to determine whether percutaneous techniques can also be used to facilitate ruptured AAA repairs and impact perioperative care. Overall, we observed that rPEVAR had essentially equivalent perioperative outcomes and a low rate of groin complications. Additionally, we observed that rPEVAR provides efficient peripheral arterial access, proximal aortic control, and facilitates timely closure of the femoral artery cannulation sites.

Traditional groin cutdown for femoral artery exposure and cannulation is not begnin (16, 17). Previous series report non-negligible rates of perioperative groin complications such as infection, hematoma, seromas, and lymphoceles (18–20). Totally percutaneous AAA repair using suture-mediated closure devices were developed with the intent to reduce groin complications and allow for a shorter postoperative recovery period. Initial experience with the PerClose ProStar XL device 3–0 braided polyester suture suggested a potential risk of infection, and even a slightly higher risk of lower extremity amputation and death (21–23). Subsequent generation devices such as the PerClose ProGlide device using a 3–0 polypropylene sutures was found to have lower risk of perioperative infection, shorter operative time (24–26), and earlier postoperative ambulation (25). Compared to EVAR performed *via* open femoral exposure, PEVAR using the Perlose ProGlide device demonstrated reduced intraoperative blood loss (27), lower limb complications (11, 13–15), and shorter hospital stays (14, 28).

There are several technical lessons from elective PEVAR that are directly transferable for successful rPEVAR. Al-Khatib et al. reported that percutaneous femoral artery cannulation was safest when performed using ultrasound guidance (26). Additionally, they reported that patients with dense femoral wall plaque was a risk factor for failed preclose closure since the femoral plaque may be inadvertently engaged by the foot plate of the closure device and cause suture mal-deployment (8, 13). Accordingly, in patients who received a preoperative CTA, careful inspection of the femoral arteries can help determine the burden of disease within in the ilio-femoral arterial segment. The majority of our patients were able to receive a CTA either prior to transfer to our facilities, or in the emergency department immediately upon arrival. Recent Society for Vascular Surgery guidelines recommend that radiologic confirmation with CT is only required when alterative diagnoses are more likely on clinical grounds (29). In our facilities CTA was deferred in patients with severe hemodynamic compromise and shock (inotropic support, blood transfusions, and active resuscitation). This included 4 patients who were immediately transferred upon arrival from the emergency department to the operating room for percutaneous placement of aortic occlusion balloon from a femoral cannulation followed by an aortogram to determine EVAR candidacy and feasibility.

Prior PEVAR experience also informs us that routine and careful ultrasound-guided inspection of the bilateral femoral arteries is necessary for successful PEVAR (8, 13, 14). As such we routinely performed this for all ruptured AAA patients as soon as the they were transferred onto the operating room table. Careful ultrasound inspection can once again help determine the burden of atherosclerotic disease in the femoral artery, identify areas along the ventral and posterior wall of the femoral artery that have the least amount of occlusive disease, and help rule out aberrant anatomy and/or high femoral bifurcations. Once the patient abdomen and groins are treated with sterile prep, but has not yet received general anesthesia, a local anesthetic is administered to the bilateral groins overlying femoral arteries. Small 1 cm–2 cm skin incisions are made in the groins and blunt finger dissection is used to generate a subcutaneous track down to the femoral artery. Ultrasound guidance is then to cannulate the most favorable segment of the femoral artery in each groin.

In our case series we observed that ultrasound-guided femoral artery cannulation was feasible in all patients, including in 9 patients who had 50%–75% femoral artery stenosis. In all patients, early cannulation of the femoral arteries facilitated early sheath advancement into the infrarenal aorta, and advancement of a suprarenal aortic occlusion balloon to provide proximal aortic control. In one patient after completion of successful EVAR an open femoral cut down exposure of the contralateral groin was performed to facilitate an open repair due to severe intraluminal occlusive disease. Therefore, this experience informs us that even in patients with severe femoral artery occlusive disease rPEVAR is feasible, with the expectation that subsequent open femoral cut down may be necessary after EVAR to treat the underlying femoral artery disease.

Survival of ruptured AAA patients depends on timely control of the aortic hemorrhage and seal of the aortic rupture site. Berland et al. report methods for sequential placement of a suprarenal aortic occlusion balloons during deployment of an EVAR mainbody and bilateral iliac limbs (30). PEVAR is uniqly suited to facilitate this technique since following placement of crisscrossing Perclose ProGlide device sutures, a 12 French sheath that is at least 45 cm long can be advanced from the contralateral groin over a stiff wire into the pararenal aorta. Through the sheath an aortic occlusion balloon is then advanced to the suprarenal aortic segment, inflated and maintained into place by the long sheath. At this point we often then induced general anesthesia, allowed for additional resuscitation measures, and carefully monitored patient hemodynamics. Once the patient is stabilized the EVAR mainbody is then advanced through the ipsilateral groin and deployed under fluoroscopy in the infrarenal aorta. Through the ipsilateral groin another aortic balloon is then advanced into the infrarenal aortic neck segment and inflated. The suprarenal balloon is deflated and removed to facilitate contralateral gate cannulation and deployment of the contralateral iliac limb. At this point the aortic occlusion balloon is deflated and removed to facilitate deployment of the ipsilateral iliac limb. Our experience demonstrated that this technique is highly effective in maintaining patient hemodynamic stability and limiting rupture-associated hemorrhage during the sequential EVAR procedural steps.

We acknowledge some limitations in our study. Given the retrospective nature of the study and limited study sample size, we expect that there were patient and procedural confounders that could not be accounted for in our analysis. Additionally, historical controls were all treated with the Endologix AFX device (9), whereas our contemporary cohort were treated with a diverse group of aortic endografts including (Gore Excluder, Cook Zenith, and Medtronic Endurant devices). Contralateral sheath size for AFX devices usually only requires a 7 French sheath size, whereas all other devices require a contralateral sheath size of at least 12 French. Therefore differences in types of aortic endografts used, and methods for stent advancement and deployment could have affected our procedural times. In addition, we did not collect femoral access time at the initiation of rEVAR or rPEVAR and the time it took to advancement a sheath into the aorta. Nevertheless, we believe our findings provide novel insights in feasibility of PEVAR for ruptured AAA, and provide the impetus for further investigation of this technique in larger clinical cohorts.

We observed that totally percutaneous EVAR for ruptured AAA (rPEVAR) is feasible, effective, and with comparable outcomes to historical elective PEVAR outcomes. rPEVAR was associated with only a few groin compilations and only two cases required an open groin cut down conversion for femoral artery repair following EVAR. We anticipate that use of such procedural adjuncts may continue to improve outcomes in critical ruptured AAA patients.

## Data Availability

The original contributions presented in the study are included in the article/**Supplementary Material**, further inquiries can be directed to the corresponding author.

## References

[1] Vardulaki KA Prevost TC Walker NM Day NE Wilmink AB Quick CR Growth rates and risk of rupture of abdominal aortic aneurysms. Br J Surg. (1998) 85(12):1674–80. 10.1046/j.1365-2168.1998.00946.x 9876073

[2] Volodos NL. Historical perspective: the first steps in endovascular aortic repair: how it all began. J Endovasc Ther. (2013) 20(Suppl 1):I-3–23. 10.1583/1545-1550-20.sp1.I-3 23448181

[3] Lindholt JS Henneberg EW Fasting H. Decreased mortality of abdominal aortic aneurysms in a peripheral county. Eur J Vasc Endovasc Surg. (1995) 10(4):466–9. 10.1016/S1078-5884(05)80170-4 7489216

[4] Berridge DC Chamberlain J Guy AJ Lambert D. Prospective audit of abdominal aortic aneurysm surgery in the northern region from 1988 to 1992. Northern vascular surgeons group. Br J Surg. (1995) 82(7):906–10. 10.1002/bjs.1800820716 7648104

[5] Bengtsson H Bergqvist D. Ruptured abdominal aortic aneurysm: a population-based study. J Vasc Surg. (1993) 18(1):74–80. 10.1067/mva.1993.42107 8326662

[6] Parodi JC Palmaz JC Barone HD. Transfemoral intraluminal graft implantation for abdominal aortic aneurysms. Ann Vasc Surg. (1991) 5(6):491–9. 10.1007/bf02015271 1837729

[7] Haas PC Krajcer Z Diethrich EB. Closure of large percutaneous access sites using the prostar XL percutaneous vascular surgery device. J Vasc Surg. (1999) 6(2):168–70. 10.1177/152660289900600209 10473335

[8] Dosluoglu HH Cherr GS Harris LM Dryjski ML. Total percutaneous endovascular repair of abdominal aortic aneurysms using perclose ProGlide closure devices. J Endovasc Ther. (2007) 14(2):184–8. 10.1177/152660280701400210 17484534

[9] Nelson PR Kracjer Z Kansal N Rao V Bianchi C Hashemi H A multicenter, randomized, controlled trial of totally percutaneous access versus open femoral exposure for endovascular aortic aneurysm repair (the PEVAR trial). J Vasc Surg. (2014) 59(5):1181–93. 10.1016/j.jvs.2013.10.101 24440678

[10] Lee WA Brown MP Nelson PR Huber TS Seeger JM. Midterm outcomes of femoral arteries after percutaneous endovascular aortic repair using the preclose technique. J Vasc Surg. (2008) 47(5):919–23. 10.1016/j.jvs.2007.12.029 18328666

[11] Lee WA Brown MP Nelson PR Huber TS. Total percutaneous access for endovascular aortic aneurysm repair (“preclose” technique). J Vasc Surg. (2007) 45(6):1095–101. 10.1016/j.jvs.2007.01.050 17398056

[12] Bechara CF Barshes NR Pisimisis G Chen H Pak T Lin PH Predicting the learning curve and failures of total percutaneous endovascular aortic aneurysm repair. J Vasc Surg. (2013) 57(1):72–6. 10.1016/j.jvs.2012.07.050 23127982

[13] Bent CL Fotiadis N Renfrew I Walsh M Brohi K Kyriakides C Total percutaneous aortic repair: midterm outcomes. Cardiovasc Intervent Radiol. (2009) 32(3):449–54. 10.1007/s00270-009-9537-3 19296162

[14] Jean-Baptiste E Hassen-Khodja R Haudebourg P Bouillanne PJ Declemy S Batt M. Percutaneous closure devices for endovascular repair of infrarenal abdominal aortic aneurysms: a prospective, non-randomized comparative study. Eur J Vasc Endovasc Surg. (2008) 35(4):422–8. 10.1016/j.ejvs.2007.10.021 18166490

[15] Krajcer Z Gregoric I. Totally percutaneous aortic aneurysm repair: methods and outcomes using the fully integrated IntuiTrak endovascular system. J Cardiovasc Surg (Torino). (2010) 51(4):493–501. 10.1016/j.jcvs.2007.01.050 20671633

[16] Stelter W Umscheid T Ziegler P. Three-year experience with modular stent-graft devices for endovascular AAA treatment. J Vasc Surg. (1997) 4(4):362–9. 10.1583/1074-6218(1997)004<0362:tyewms>2.0.co;2 9418200

[17] Brewster DC Geller SC Kaufman JA Cambria RP Gertler JP LaMuraglia GM Initial experience with endovascular aneurysm repair: comparison of early results with outcome of conventional open repair. J Vasc Surg. (1998) 27(6):992–1003; discussion 1004–5. 10.1016/S0741-5214(98)70002-3 9652461

[18] Chuter TA Reilly LM Stoney RJ Messina LM. Femoral artery exposure for endovascular aneurysm repair through oblique incisions. J Vasc Surg. (1998) 5(3):259–60. 10.1583/1074-6218(1998)005<0259:faefea>2.0.co;2 9761579

[19] May J White GH Yu W Waugh RC Stephen MS McGahan T Surgical management of complications following endoluminal grafting of abdominal aortic aneurysms. Eur J Vasc Endovasc Surg. (1995) 10(1):51–9. 10.1016/S1078-5884(05)80198-4 7633970

[20] Slappy AL Hakaim AG Oldenburg WA Paz-Fumagalli R McKinney JM. Femoral incision morbidity following endovascular aortic aneurysm repair. Vasc Endovascular Surg. (2003) 37(2):105–9. 10.1177/153857440303700204 12669141

[21] Johanning JM Franklin DP Elmore JR Han DC. Femoral artery infections associated with percutaneous arterial closure devices. J Vasc Surg. (2001) 34(6):983–5. 10.1067/mva.2001.120033 11743549

[22] Cherr GS Travis JA Ligush J Jr. Plonk G Hansen KJ Braden G Infection is an unusual but serious complication of a femoral artery catheterization site closure device. Ann Vasc Surg. (2001) 15(5):567–70. 10.1007/s10016-001-0002-2 11665443

[23] Geary K Landers JT Fiore W Riggs P. Management of infected femoral closure devices. Cardiovasc Surg. (2002) 10(2):161–3. 10.1177/096721090201000213 11888747

[24] Morasch MD Kibbe MR Evans ME Meadows WS Eskandari MK Matsumura JS Percutaneous repair of abdominal aortic aneurysm. J Vasc Surg. (2004) 40(1):12–6. 10.1016/j.jvs.2004.03.019 15218455

[25] Torsello GB Kasprzak B Klenk E Tessarek J Osada N Torsello GF. Endovascular suture versus cutdown for endovascular aneurysm repair: a prospective randomized pilot study. J Vasc Surg. (2003) 38(1):78–82. 10.1016/S0741-5214(02)75454-2 12844093

[26] Al-Khatib WK Zayed MA Harris EJ Dalman RL Lee JT. Selective use of percutaneous endovascular aneurysm repair in women leads to fewer groin complications. Ann Vasc Surg. (2012) 26(4):476–82. 10.1016/j.avsg.2011.11.026 22437069

[27] Howell M Doughtery K Strickman N Krajcer Z. Percutaneous repair of abdominal aortic aneurysms using the AneuRx stent graft and the percutaneous vascular surgery device. Catheter Cardiovasc Interv. (2002) 55(3):281–7. 10.1002/ccd.10072 11870928

[28] Smith ST Timaran CH Valentine RJ Rosero EB Clagett GP Arko FR. Percutaneous access for endovascular abdominal aortic aneurysm repair: can selection criteria be expanded? Ann Vasc Surg. (2009) 23(5):621–6. 10.1016/j.avsg.2008.09.002 18954964

[29] Chaikof EL Dalman RL Eskandari MK Jackson BM Lee WA Mansour MA The society for vascular surgery practice guidelines on the care of patients with an abdominal aortic aneurysm. J Vasc Surg. (2018) 67(1):2–77.e2. 10.1016/j.jvs.2017.10.044 29268916

[30] Berland TL Veith FJ Cayne NS Mehta M Mayer D Lachat M. Technique of supraceliac balloon control of the aorta during endovascular repair of ruptured abdominal aortic aneurysms. J Vasc Surg. (2013) 57(1):272–5. 10.1016/j.jvs.2012.09.001 23159478

