# Supplementary material for: Totally percutaneous endovascular repair for ruptured abdominal aortic aneurysms
Source: Front Surg. 2022 Oct 21;9:1040929. doi: 10.3389/fsurg.2022.1040929 (PMC9634472; doi:10.3389/fsurg.2022.1040929)
Supplement: Supplementary file 1 [file Datasheet1.pdf]

Supplementary Material

1 Supplementary Tables

1.1 **Table SI.** Baseline characteristics of the patients

	**Study group** (n=35)	**ePEVAR*** (n=50)	***P***
Age (years)	72 ± 9	70 ± 6.6	*.25*
Male sex	30 (86)	47 (94)	*.27*
Height (cm)	177 ± 8	179 ± 6.9	*.40*
Weight (kg)	95 ± 22	93 ± 13	*.72*
BMI (kg/m^2)	30 ± 6	29 ± 3.9	*.46*
Smoking	13 (37)	43 (86)	***<.001***
Diabetes	10 (29)	14 (28)	*.95*
CVA	0	3 (6)	*.27*
COPD	12 (34)	15 (30)	*.68*
CHF	7 (20)	8 (16)	*.63*
CAD	16 (46)	19 (38)	*.48*
Prior PCI	4 (11)	10 (20)	*.38*
HTN	30 (86)	42 (84)	*.83*
HLD	19 (54)	45 (90)	***<.001***
MI	2 (6)	6 (12)	*.46*
Renal failure	0	1 (2)	*1.00*
TAA	1 (3)	0	*.41*
Pre-operative shock	17 (49)	n/a	-

*ePEVAR, elective percutaneous EVAR from PEVAR trial; rPEVAR, ruptured percutaneous EVAR; rEVAR, ruptured EVAR with femoral cutdown; BMI, body mass index; CVA, cerebrovascular accident; COPD, chronic obstructive pulmonary disease; CHF, congestive heart failure; CAD, coronary artery disease; PCI, percutaneous coronary intervention; MI, myocardial infarction; TAA,

thoracic aortic aneurysm; n/a, data not available. Data are presented as number (%) for categorical variables and mean ± standard deviation for continuous variables.

1.2 Table SII. Perioperative and short-term outcomes

	**Study group** (n=35)	**ePEVAR*** (n=50)	***P***
Procedural and in-hospital outcomes			
Procedure time (mins)	157 ± 73	107 ± 45	***.04***
Blood transfusion	22 (63)	4 (8)	***<.001***
ICU length of stay (hrs)	113 ± 150	26 ± 9.0	***.004***
Hospital stay (days)	14 ± 23	1.3 ± 0.7	***.002***
Major adverse events at 30 days			
30-day MAE	12 (34)	3 (6)	***.001***
Death	2 (6)	0	*.17*
Conversion to open repair	2 (6)	0	*.17*
Bowel ischemia	2 (6)	0	*.17*
Cardiac morbidity	3 (9)	0	*.07*
Neurologic complication	3 (9)	0	*.07*
Renal failure	4 (11)	2 (4)	*.22*
Respiratory complication	5 (14)	1 (2)	*.08*
Secondary procedure	8 (23)	0	***<.001***

*ePEVAR, elective percutaneous EVAR from PEVAR trial; rPEVAR, ruptured percutaneous EVAR; rEVAR, ruptured EVAR with femoral cutdown; ICU, Intensive Care Unit; MAE, major adverse events. Data are presented as number (%) for categorical variables and mean ± standard deviation for continuous variables.

1.3 Table SIII. Femoral access-site techniques and complications

	**Study group** (n=35)	**ePEVAR*** (n=50)	***P***
Procedural Access Technique			
Successful Pre-close	16 (46)	4 (8)	***<.001***
Pre-close conversion to cutdown	2 (6)	0	*0.17*
Femoral Access-Site Complications at 30 days			
30-day FAAC	10 (29)	6 (12)	*.054*
Arteriovenous fistula	0	0	-
Femoral neuropathy	0	0	-
Hematoma	1 (3)	0	*.41*
Dissection	3 (9)	0	*.07*
Infection	4 (11)	0	***.026***
Lymphocele	0	0	-
Thrombosis/occlusion	0	2 (4)	*.51*
Vascular injury	0	1 (2)	*1.00*
Lower extremity ischemia	1 (3)	2 (4)	*1.00*
Bleeding/transfusion	4 (11)	1 (2)	*.15*

*ePEVAR, elective percutaneous EVAR from PEVAR trial; rPEVAR, ruptured percutaneous EVAR; rEVAR, ruptured EVAR with femoral cutdown; FAAC, Femoral Artery Access Complications. Data are presented as number (%) for categorical variables and mean ± standard deviation for continuous variables.
